# Technological advances in vitiligo management: perspectives on AI, mobile tools, and clinical utility

**DOI:** 10.3389/fmed.2025.1661554

**Published:** 2025-10-06

**Authors:** Manasi Parikh, Gloria Fang, Faith Poon, Manuella Kyeremeh, Daniel Cruz, Karen Ki, Sooyee Huang, Siya Lin, Collin Hong, Hannah O. Chan

**Affiliations:** ^1^McMaster University Department of Family Medicine, Hamilton, ON, Canada; ^2^Skinopathy Research, North York, ON, Canada; ^3^The University of Buckingham Medical School, Crewe, United Kingdom; ^4^University of Toronto Temerty Faculty of Medicine, Toronto, ON, Canada; ^5^Western University Faculty of Science, London, ON, Canada; ^6^Scarborough Health Network Centenary Hospital, Scarborough, ON, Canada

**Keywords:** vitiligo, AI, technology, healthcare, dermatology

## 1 Introduction

Vitiligo is a chronic skin disorder characterized by the selective loss of melanocytes, resulting in the development of depigmented patches on the skin (1). Recent epidemiological studies estimate the global prevalence of vitiligo to be approximately 0.36–0.40%, with higher prevalence in adults than children, and notable geographical variation (2, 3). Although the past decade of research has established vitiligo as a disease of autoimmune origin, the underlying pathogenic mechanisms are not fully understood, and no definitive cure currently exists (4, 5). Furthermore, new areas of research, such as the relationship between the skin and gut microbiome, are emerging; however, the data remain inconclusive due to variability in methodologies and sample sizes (6). Despite advances in therapeutic approaches, vitiligo patients often suffer from delayed diagnosis due to a scarcity of dermatological resources and expertise (7, 8). Since many vitiligo therapies are most effective in the early stages of disease (9, 10), this diagnostic gap underscores the need for innovative solutions that can enhance the accuracy and accessibility of clinical care.

In recent years, technological innovations utilizing artificial intelligence (AI) and computer vision models have begun to reshape the dermatological landscape, offering the potential to enhance diagnostic precision and support clinical decision-making (11). These tools hold particular promise for vitiligo, where the monitoring of disease progression and treatment response has historically been difficult to standardize (12). Recently, Abdolahnejad et al. proposed an AI-driven mobile application that enables patients to remotely assess and track the progression of their vitiligo (13). While this approach is encouraging, it also invites questions about clinical utility, generalizability, and readiness for integration into routine care. It is also necessary to consider the broader clinical applications of vitiligo tools, such as their potential role in psychosocial support and holistic patient care (14, 15). In this opinion paper, we place this proof-of-concept study in the context of the broader technological landscape for vitiligo, discussing both emerging and established tools, and reflecting on their potential to improve clinical outcomes and access to care. Here, we define *clinical utility* to mean a demonstrated, measurable benefit to patient care or workflow (e.g., improved diagnostic agreement, reduced time-to-treatment, or more reliable VASI/T-VASI change detection). We define *generalizability* to mean stable performance across Fitzpatrick skin types I–VI, anatomic sites, capture contexts (clinic cameras and mobile), and across care settings (specialist, primary care, and teledermatology).

## 2 Existing vitiligo tools

Vitiligo is primarily diagnosed through clinical examination, based on the presence of white macules with or without depigmented hairs (leukotrichia) in affected areas of the skin (1, 16). Diagnosis can be supported by Wood's lamp examination, dermoscopy, punch biopsy, and molecular testing to rule out other causes of hypopigmentation (10, 17). The most common tool used to assess vitiligo is the Wood's lamp, which emits ultraviolet (UV) light at wavelengths between 320 and 400 nm, with a peak at 365 nm (10, 18). Under the Wood's lamp, vitiligo appears as bright bluish-white patches, often with sharp demarcations (18, 19). This enables clinicians to detect subtle depigmentation in fair-skinned individuals and identify spreading vitiligo that may not be visible in natural light (17, 18). Therefore, in addition to its diagnostic value, the Wood's lamp can also be used to non-invasively monitor active disease progression and repigmentation after treatment (17, 20).

Vitiligo assessment and monitoring can also be supported by other imaging modalities, including dermoscopy and reflectance confocal microscopy (RCM) (21, 22). Though less commonly used, RCM is primarily a diagnostic technique used to visualize the epidermis and superficial dermis at near-histological resolution (23). Although RCM can also support monitoring of disease activity and treatment response, its clinical use is limited by the need for specialized equipment and training (24–26). In contrast, dermoscopy provides clinicians with an illuminated, magnified view of the epidermis and papillary dermis, enabling visualization of abnormalities in the pigmentary network, as well as perifollicular and perilesional changes (27, 28). While some dermoscopic features used to differentiate stable from unstable vitiligo are inconsistently reported in the literature (27–30), a recent study has found that dermoscopy and Wood's lamp are equally helpful, with dermoscopy demonstrating a slightly higher agreement with clinical assessments (31). However, both methods only demonstrate fair agreement (κ = 0.33 for Wood's lamp and κ = 0.40 for dermoscopy) with clinical evaluations (31). Therefore, correlating dermoscopic findings with other tools may improve diagnostic accuracy and clinical confidence (21, 22).

Traditionally, vitiligo progression and treatment response are evaluated by combining photographic documentation with clinical scoring systems (10, 17). Common systems that assess the size and extent of depigmentation include the Vitiligo Area Scoring Index (VASI), its facial (F-VASI) and total body (T-VASI) variants (32–34), the Vitiligo European Task Force assessment (VETFa) (35), and the Vitiligo Extent Score (VES) (36, 37). Although these systems are validated and generally considered reliable, they are inherently subjective and susceptible to inter-observer variability (17, 35, 38). Proper assessment can also be time-consuming in clinical practice, and dependent on the expertise of the assessor (17). Therefore, effective vitiligo management is contingent on access to clinicians with the tools and expertise to monitor the condition.

## 3 Artificial intelligence and vitiligo

In practice, the diagnosis and treatment of vitiligo are often limited by healthcare system constraints, cost barriers, and a shortage of specialists (7, 39). As a result, there is a growing need for data-driven technologies that can provide accurate and objective assessments for vitiligo patients. These innovations are especially important for advancing equitable care in geographically remote or underserved populations.

To address these challenges, many researchers have begun exploring artificial intelligence as a means of supporting vitiligo diagnosis and monitoring. Although machine learning and artificial intelligence have only recently gained widespread prominence, predictive artificial neural network (ANN) models for vitiligo were described as early as 2009 (40). Over the past decade, deep learning models, such as convolutional neural networks (CNNs), have become central to many high-performing dermatological AI systems (41–44). These multi-layered systems are trained on large datasets of labeled images, and can automatically extract key hierarchical features to make predictions (42, 45).

The architecture of a CNN determines how its layers are arranged and connected, and it plays a critical role in how a model processes images and generates outputs (45, 46). In the context of vitiligo, deep learning models are typically designed to either identify vitiligo (classification) or measure and quantify depigmentation (segmentation). Depending on the task, modern CNN models often adopt architectures from the ResNet, DenseNet, EfficientNet, YOLO, Inception, and U-Net families (43, 45, 46). These model families are most clinically useful when mapped to: (i) differential diagnosis (vitiligo vs. mimickers such as pityriasis alba or tinea versicolor), and (ii) burden quantification to complement VASI/T-VASI for treatment decisions. Segmentation models directly support serial burden tracking and re-pigmentation analysis, while detection models help standardize photography by localizing lesions and proposing consistent fields of view for follow-up.

For classification tasks, ResNet (42, 47), DenseNet (42, 48), EfficientNet (13, 47), and Inception (49, 50) architectures have been successfully applied by various groups to distinguish vitiligo from other skin conditions and/or healthy skin. These architectures are typically responsible for feature extraction and serve as the backbone of a CNN model. In contrast, architectures such as U-Net (51), PSPNet (52), and fully convolutional neural networks (FCNNs) (53) have been used to perform segmentation tasks for vitiligo lesions. Traditional machine learning algorithms, including K-means clustering, have also been used in combination with deep learning systems for vitiligo segmentation (13, 54). Practically, backbones support diagnosis vs. mimickers, segmentation nets support extent/re-pigmentation tracking, and detection models support standardized image capture for longitudinal care.

Architectures in the You Only Look Once (YOLO) family are object detection frameworks that can be adapted for vitiligo lesion classification and localization (52). Instead of performing classification on entire images, YOLO models can be trained to rapidly detect and draw bounding boxes around skin lesions (55). In 2023, Guo et al. proposed a hybrid deep learning model for vitiligo that integrated the YOLO v3 architecture for lesion detection and a U-Net++ architecture for segmentation (52). This system achieved a lesion detection sensitivity of 92.91% and a segmentation Jaccard index of 0.79 (52). Clinically, fast lesion localization can improve site selection for phototherapy and enable consistent recapture of vitiligo lesions across visits.

While CNN-based systems remain the foundation of most current models, recent advances in transformer-based architectures suggest new possibilities for even greater performance and generalizability (56, 57). Transformers are deep learning architectures that use self-attention mechanisms to model complex relationships within input data (57). In 2024, Zhong et al. demonstrated that shifted window (Swin) transformers outperformed CNN ResNet models in the classification of vitiligo, with top accuracies of 93.82% vs. 89.26% (58). For clinicians, these systems may translate into improved early change detection (e.g., perifollicular repigmentation) and potential **“**second-reader**”** support in uncertain cases.

Overall, deep learning systems reported in the literature consistently demonstrate strong performance, with classification accuracies ranging from 66% to 97% (47, 50) and segmentation accuracies between 93% and 97% (54, 59). Notably, the model developed by Liu et al., which reported the lowest classification accuracy (66%), was found to be non-inferior to dermatologists and outperformed both primary care physicians and nurse practitioners in diagnostic accuracy (50). These direct comparisons with healthcare professionals underscore the clinical relevance of deep learning systems for vitiligo assessment and highlight their potential to enhance the accessibility and consistency of vitiligo care.

## 4 AI applications for vitiligo patients

Although research into dermatological AI is rapidly advancing, few models have successfully translated into practical clinical or patient-facing applications (Table 1). This slow adoption is largely due to valid concerns around data privacy, trust, regulatory hurdles, and the need for robust external validation (60, 61). In practice, patient-facing tools can support self-monitoring, enable teledermatology triaging, and offer standardized photography to improve the reliability of detecting changes. While some mobile applications allow users to upload images and manually track their vitiligo, there are currently no widely adopted tools that leverage AI to quantitatively monitor disease progression.

**Table 1:** Landscape of vitiligo applications.

**Name**	**Purpose**	**AI features?**	**User feedback?**	**Patient support tools?**	**Available?**
Vitiligo diagnostic assistance (Vi-DA) (62)	Vitiligo lesion segmentation	**√**	**√**		
MiDerm (65)	Informational, behavioral, and peer support for patients		**√**	**√**	
Skinopathy vitiligo (13)	Vitiligo lesion segmentation and tracking	**√**			**√**

One of the earliest patient-facing tools described in the literature is *Vitiligo Diagnostic Assistance (Vi-DA)*, a prototype Android application introduced by Nugraha et al. in 2018 (62). Vi-DA was developed using traditional image processing algorithms and unsupervised machine learning for the segmentation of vitiligo lesions. The segmentation algorithm employed Fuzzy C-Means (FCM) clustering and yielded similar results to alternative segmentation models (62). However, formal performance metrics were not reported, and evaluations were limited to qualitative comparisons with images captured under normal lighting. Notably, the authors also assessed the usability of Vi-DA with vitiligo patients, who expressed generally positive feedback, but concerns about long processing times (62). Despite this early exploration, the Vi-DA application remained at the prototype stage and was never released for public use.

More recently, Abdolahnejad et al. introduced a proof-of-concept mobile application that applies deep learning techniques to detect and monitor vitiligo (13). The AI system is embedded in a mobile interface and performs classification, segmentation, and colorimetric analysis on user-submitted images. Vitiligo detection is carried out by a CNN based on the EfficientNet-B7 architecture, which achieved a reported accuracy of 95.0% (13). Following lesion identification, segmentation is performed using K-means clustering and Boundary Attention Mapping (BAM), a technique that utilizes activation maps from the CNN to refine lesion boundaries (13, 63). The system also performs colorimetric analysis on segmented regions by extracting pixel-level color data, providing users with an assessment of vitiligo severity and depigmentation. While quantitative performance metrics for segmentation and colorimetry were not reported, qualitative comparisons between AI segmentations and images taken under the Wood's lamp demonstrated close visual alignment (13).

Despite promising technical results, further large-scale clinical validation studies are needed to evaluate the generalizability of these deep learning systems. These studies can offer valuable insights into the performance of AI models across diverse patient populations, skin tones, and lighting conditions (64). As interest in AI-based tools for vitiligo continues to grow, future work should address both technical improvements and broader considerations for clinical adoption. Implementation strategies and mechanisms for clinician oversight will also be essential to support the safe and effective use of AI tools in real-world settings.

While AI-based vitiligo applications primarily focus on clinically relevant features, it is equally important to recognize the value of holistic digital tools that support patients beyond the clinic. One proposed tool is MiDerm, which is an application that provides education, self-management resources, and peer support for patients living with different skin conditions, including vitiligo (65). Although the application is currently under development, preliminary qualitative studies have highlighted the significant psychological burden associated with vitiligo and the unmet need for additional support (65). By targeting aspects of quality of life that extend beyond physical symptoms, these tools highlight the value of integrating medical and psychosocial support into digital platforms. Therefore, the most impactful patient-facing applications for vitiligo will combine clinically useful AI functionalities with holistic support features, offering a comprehensive approach to disease management.

## 5 Recommendations for future applications

Although promising AI tools for vitiligo are emerging, few have undergone the rigorous development, validation, and integration necessary for widespread clinical adoption. To ensure that these technologies can effectively support patient care and meet the standards of clinical practice, future efforts should prioritize strategies that address the needs and concerns of both clinicians and patients. The following recommendations highlight key considerations that may help guide the development of future vitiligo AI applications.

I. **Clinical validation:** large-scale clinical studies are essential to evaluate the effectiveness of AI applications in routine practice. Performance should be assessed not only in comparison to dermatologists but also across different care settings and patient demographics. Utility should be reported by task-specific endpoints (e.g., ΔVASI minimal detectable change, triage accuracy vs. dermatologist, and time-to-treatment) and generalizability should be demonstrated through pre-specified analyses across various Fitzpatrick skin types (I–VI), devices, lighting conditions, and anatomic sites. The inclusion of outcome metrics such as diagnostic accuracy, user adherence, and patient-reported satisfaction will improve credibility and clinical relevance.
II. **Ethical considerations and data security:** applications should adhere to stringent data protection protocols and explicitly address ethical concerns. This includes compliance with local privacy regulations, as well as transparent policies on data ownership and informed consent.
III. **Scalability and integration:** for clinical adoption, the application should integrate into the existing digital health infrastructure. This may include compatibility with electronic medical record (EMR) systems, interoperability with clinical workflows, and support for remote care models. Practical guidance for real-world implementation should be considered early in the development process. These would include device/lighting robustness checks, guidance for image quality controls, and EMR/teledermatology integration hooks (e.g., Fast Healthcare Interoperability Resources (FHIR) data formatting standards and VASI fields) to ensure performance transfers from research to practice.
IV. **User-centered design:** applications should prioritize patient usability through iterative user testing, intuitive design, and accessibility features. Incorporating structured feedback from diverse patient populations will improve engagement, adherence, and trust in the system. The evaluation of interface design alongside clinical performance is critical for real-world adoption.

## 6 Limitations and future directions

While AI holds significant promise for the future of dermatological care, it is essential to acknowledge the limitations of machine learning models. Data availability is the most obvious obstacle. Deep learning systems are highly dependent on the quality and diversity of the data they are trained on. However, many research groups rely on relatively small, homogenous datasets that do not reflect the broad spectrum of real-world patients. To address this gap, the Diverse Dermatology Images (DDI) dataset was created in 2022 to provide researchers with a publicly available, curated dataset with diverse skin tones (64). When three state-of-the-art dermatology models were tested on the DDI dataset, performance declined significantly—ROC-AUC scores dropped from ranges of 0.88–0.94 to 0.56–0.67 (64). Furthermore, in the context of vitiligo, even commonly used clinical tools such as the Wood's lamp have been reported to produce false-negative results in darker skin tones (12). Therefore, both the diversity of training datasets and the clinical benchmarks used for evaluation must be carefully considered during model development. Future studies should prioritize endpoints that reflect clinical utility and include stratified analyses that demonstrate generalizability across skin tones and image capture conditions.

It is also important to recognize the technical limitations of image-based detection and segmentation systems. As with clinical photography, standardized image capture procedures are essential for accurate tracking of vitiligo by machine learning models. Controlling factors such as lighting, positioning, and background contrast enable both clinicians and AI models to collect comparable images over time (66). The two-dimensional nature of images presents additional challenges, as AI systems often struggle to assess lesions on curved or complex anatomical surfaces (67). Natural artifacts, such as hair or shadows, can further interfere with model performance (67). However, emerging solutions, such as 3D imaging, image preprocessing techniques, and calibration tools, may help address these limitations (67, 68).

Ultimately, the advancement of AI for vitiligo will depend not only on technological improvements but also on ethical and inclusive implementation. Future models should be trained on large and diverse datasets that reflect variations in skin tone, age, and anatomical location. The inclusion of quality-of-life measures in patient-facing applications can also support a more holistic approach to vitiligo care. When designed with clinical and patient-centered perspectives in mind, AI has the potential to enhance diagnosis, support early intervention, and promote equitable care for individuals living with vitiligo. Realizing this potential will require close collaboration between researchers, clinicians, patients, and policymakers to ensure that AI tools are accurate, accessible, and trustworthy.
